# Re: Can rectal catheters be avoided during paediatric urodynamic studies? By Abhilash Cheriyan, Arun Jacob Philip George, Antony Devasia and J. Chandrasingh

**DOI:** 10.1080/2090598X.2019.1694763

**Published:** 2019-12-04

**Authors:** Fawzy Farag

**Affiliations:** Sohag University Hospital, Sohag, Egypt, ff.farag@gmail.com

Dear Editor,

This was a retrospective study of a paediatric group of patients with lower urinary tract dysfunction of various underlying pathology. The aim of the study was to determine the reliability of single-channel urodynamics study using a single intravesical pressure sensitive catheter to detect some imperative urodynamic parameters including low bladder compliance and detrusor overactivity. And moreover, the authors used pressure–flow curves in conjunction with urinary flow curves to determine voiding with straining patterns. The ultimate goal was to determine the feasibility of conducting urodynamic investigations in paediatric populations in the future avoiding the use of a rectal catheter, which adds to the child’s distress during the relatively invasive investigation [1]. This has been addressed in previous studies on adult populations [2,3]. The rationale behind the study is plausible and worthy of investigation indeed. However, the methodology followed by the authors of the current study is suboptimal for the following reasons. Firstly, it was a retrospective study; such an interesting idea requires a prospective controlled blinded approach. Secondly, the sample size is small and the study is not sufficiently powered, which is important to justify the significance of the statistical outcomes of the study. In addition, the whole study relies totally on the experience of a single investigator, which makes it very subjective and operator dependent, and consequently the study is lacking important variables including the intra-observer and inter-observers’ agreements. Therefore, the reproducibility and accuracy of the study could be questionable.
